# His Gift

**Published:** 1910-03

**Authors:** Roy Norton


### BUFFALO MEDICAL JOURNAL.

Vol. Lxv. MARCH, 1910. No. 8

### ORIGINAL COMMUNICATIONS.

### His Gift

By ROY NORTON.

(From Sunday Magazine of the *New York Tribune*, December 12, 1909.)

*NOTE.—We offer no apology for printing in these columns a story out of the scientific groove. The hero is a real character now living in the mountainous region of New Jersey where he has practised many years. Our readers will recognise him as a type of physician now rapidly disappearing.—Ed. B. M. J.*

IF you had lived in those mountains that shield the mainland of our country from the Atlantic coast line, you might have met him. Perhaps it would have been on some dark night when the trails through the wilderness were dim, and you, terrified by the mystery of Nature in her black cloak, had paused with straining ear to catch an unusual sound. A slow, stumbling noise would become more clearly audible, and from your position at the side of the trail where vine and thicket joined you would see a shape loom up against the stars above you, a shape of man and horse, the former huddled over with folded arms and bent head.

If the horse didn't start and betray your presence to his rider, they two would pass off into the shadows, moving steadily toward some distant goal, and you would for the first time have met Dr. Harvey—and would later know that the flame of his great spirit, exhausted, had burned low through physical weakness and that, having brought ease to others, he slept as tired soldiers sleep, in the saddle.[1] That the horse passed slowly was proof that the battle was over; for had it been the call to meet that universal enemy, Pain, you might have found scant time to leave the road, else that charger with back-laid ears, lathering flank, and steaming breath had run you down.

If he had been pointed out to you in some wayside tavern of the wild, the man who did it would have whispered his name reverentially, with some crude apology for his deference, as befits free men of the mountains when admitting that before them stands a superior. It might go something like this, in that free speech of the forest:

"That's Doc Harvey, him as saved my wife; who told the president of the powder works to his teeth that he'd been careless

1. Reprinted by special permission of the author.

of his men when the old Number Seven magazine went up; who cries when he fights, but is unafraid; who cries when somebody's baby dies; who lends his money or his life to us who stand in need."

THE man who was unafraid would take a new semblance to your critical eyes, and you would stare at him as he stood there in the half-lighted taproom giving instructions to the stable boy and watching the landlord dip the hot poker into the steaming ale. A tall man he was and, at first, plain to look upon, thin, angular, smooth shaven, and not overly strong of frame. If talking, his voice came from the frail shell of his throat and body with a melancholy boom, decisive, and with an under note of power. If he turned toward you with that sharp, steady look from blue-gray eyes, he was transformed.

You saw a soul, and it was brave. It analysed you and your worth in one sweeping inventory; not by that worth of dollars by which the tax collector regulates his obeisance, but by that standard which asks if your conscience rings true as the bell that is cast without flaw. And the longer you know him the more you understood that his was a heart of courage.

He would stop a man of wealth in the road and lash his petty soul with the scourge of his tongue did he abuse a horse, or he would lean above the couch of the dead as tenderly as a mother whispering prayers over her dying young. He had no enemies save the undertakers; yet he would seize a ruffian by the throat, or, so the countryside whispered, use methods of his own with hysterical women.

For instance, there was Bill Harmsworth's wife, a woman who loved cheap frills and sweeping city veils and used to throw herself on the floor and shriek when her husband declined to mortgage the home to give her a trip to the seaside. When Dr. Harvey was called he sent William away and used some unsympathetic words and the back of a hair-brush, assuring the patient that the next time he came he would bring a board. And, strange to relate, she never after had hysteria, and the doctor was never again required.

He roundly and faithfully spanked Tom Minion's boy for tying a can to a stray dog's tail, and an hour afterward dived into the lake and rescued the youngster from drowning while his fellow urchins howled upon the bank. He slapped big Casey, the woodcutter, for speaking ill of the dominie, told him he was the scum of the earth, and within the week nursed the same Casey like a woman when a falling tree malevolently crushed his leg. That he should champion the dominie was not, most of us in the hills believed a confession of religious tendency; but rather a declaration of faith in the preacher as a man. Indeed, no one knew whether Dr. Harvey possessed any other religion than that of his own broad humanity, until that Christmas Eve of—well, never mind the year; it wasn't so long ago.

THAT was the year too when we began more fully to appreciate his reputation as a professional man, although no one could have convinced us, who had known him for twenty years, that he was not the finest physician and surgeon that ever bestrode a horse. The city had encroached upon our mountains, although we were more than a hundred rugged miles away. Its millionaires had bought great tracts of land and lake and hedged them round with restrictions and warded them with gamekeepers, mostly fellows who wore corduroy breeches and couldn't shoot.

In the hearts of these reservations a few palaces had sprung up like feudal castles of which we read when boys. The telephone had stretched its webs here and there through the forests, and a railway had come so close that on still nights the shrieks of its locomotives could be heard. The roads had been improved until the mountain children no longer ran affrighted at sight of a puffing motor car, and the old stone mill at the forks, where once, according to tradition, General George Washington had paused to chuck the miller's pretty daughter 'neath her chin, had now become a garage.

None of us ever quite knew what it was about; but Dr. Harvey, we learned afterward by chance, had written a series of articles concerning some special study he had made, and these, printed in the medical journals, caused his name to be mentioned by members of his profession over all the world. For the first time since we had known him he would be absent for a day or so at a time. He had been seen at the railway station with his old slouch hat wearing a less careless crease and his coat carefully brushed. That alone was sufficient to cause comment. Everyone thought, perhaps, he was going to marry some one, despite the fact that for at least fifteen years we had done our best to interest him in some of our daughters.

You can imagine the shock of surprise, therefore, when we learned that the big medical societies of New York had called Dr. Harvey—our doctor!—down there to lecture to them; "To show them city fellers how to do things," Uncle Jess Slack asserted, and possibly he wasn't so far from the truth.

THEN came another startling piece of gossip. One of the millionaires who had a gray stone castle on Dows Peak, Dr. Butcher, had been told by the eminent city surgeon, Dr. Bull, that there was no need of his coming to New York to be treated, because within call was a great man—Dr. Harvey! Think of that!

"A great man!" Moreover, this millionaire should have known that, being a doctor himself. We used to wonder why he didn't take his own medicine, because he made his money out of "Doctor Butcher's Perfect Panacea." It was pretty good stuff. We tried a bottle when Billy had the cholera; but it didn't work so well for cholera as it did for strains and bruises. Anyway, this man Butcher began to employ our doctor, and got so well that he became liberal. He gave Dr. Harvey an automobile, so he could come faster when he was called.

There are several men still living in the mountains who can remember how Dr. Harvey looked that day when Butcher's chauffeur got him into the machine out in front of the tavern and showed him how to run it. The school teacher down at the corner let school out a half-hour earlier so the children could see the sport. The machine ran up and down the road, till at last Harvey mastered it and all the chauffeur did was to sit there with his arms folded. He was considerably more dignified than the doctor. After he had gone the doctor took, in squads, all the children, and some of the rest of us who had never before been in an auto, for a ride as far as the Budds Lake Road and back. Most of us were a little frightened. The thing did go fast, all right!

Just two nights later the doctor had a call some place and wanted to go in a hurry. The machine didn't work after he got it out in the road.

"Jim," he yelled at the hostler, "get my horse and then push this thing back into the chicken house! When I've got a call I want to get there. Gray Dick beats this contraption, because he never balks."

That machine still stands in the chicken house, and I rather think Gray Dick was pleased; for he certainly had shown some signs of jealousy. We liked our doctor better with the old gray, anyway; for many a tormented body had lifted with straining ears to catch the sound of the hoofbeats when that big gelding came tearing down the road. Not that we didn't joke Harvey a little about his idle automobile, because he was a very earnest man and it warmed the heart cockles to hear him when he grew emphatic. The most common excuse he gave was that he didn't use the machine in the daytime because Gray Dick needed exercise, and at nights he left it at home because Dick knew all the roads and could take short cuts over mountain trails.

WELL, the Christmas year of which I spoke was a phenomenal one. The fall came early. The oaks turned red within a week, and the chestnuts were scarcely filled out before the frost sent them dropping, in the stillness of the woods, to the ground. The wild geese and ducks swarmed out of the north in long V-shaped strings and covered lake and sedge before the cider in our cellars had grown hard enough to make the dominie shake his head. The chipmunks deserted their summer residences in the old stone fences weeks before their chattering usually ended, and then—mystery of mysteries!—the season changed to one of warmth, and folks quoted that old saying that "A green Christmas makes fat grave-yards." It was the strangest holiday time old Pop Woodhull Bird could remember, despite his ninety-five years in the Schooleys, and when he declared it phenomenal we agreed that it was so.

The "Green Christmas" thing seemed true. Night and day Gray Dick splattered along roads that had become muddy, and Dr. Harvey began to look worried and harassed and tired. His language grew more forcible, if that was possible, and his speech was shorter than it had been; for he felt that in this one circle of the hills the lives of all our sick were in his charge. Heaven knows how he stood it; for at every place where three or four houses clustered together there was one at least that answered the summons of the weird season!

CHRISTMAS EVE came with a night that threatened rain more than snow, one of those black, heavy nights when the air is dead and everything seems waiting for something inevitable and tinged with dread. Down at the tavern where Dr. Harvey lived the big loafing room was deserted and looked more lonesome and dingy than usual. One of the oil lamps had been smoking, and the other wasn't much cleaner than its neighbor. A big glum man came in with a lantern and wanted to know if the doctor was there.

"Nope," answered the innkeeper, who was trying to solve a puzzle that he had bought to give to one of his boys, but was himself testing to be sure it could not be worked.

The man with the lantern leaned against the bar, putting a hand on the rail. "When will he be back?"

The innkeeper paused long enough to look up at him, and saw that he was in trouble. He replied, a trifle kindlier than was his wont, "Can't tell. Might be a minute, might be an hour. Old Doc Butcher has been 'phonin' down here every fifteen minutes for a long time now. They want him up there pretty bad, I reckon."

He started the puzzle again and then, as if by an afterthought, lifted his head and asked, "Anybody sick?"

"Yes."

"Better wait, then. Maybe he'll be in purty soon."

That was as far as he was capable of commiserating; but that wasn't much solace for the man with the lantern, who moved restlessly round the room and stared out into the blackness of the night as if trying to discover the doctor coming out of it. He might have gone to look for him if just then the door hadn't opened admitting the one he sought.

The innkeeper hurried to tell Harvey that the millionaire wanted him, knowing that old Butcher always paid a hundred dollars a visit, and that is considerable money in our part of the world. Besides, he knew that our doctor most always needed it. The quiet man came forward before Harvey could say anything.

"Doctor," he said, "I don't like to be ahead of other folks' sufferings; but—but—well, you see, it's my boy. He's the only one we have, and—Doctor—he's such a fine little feller and—and—" That was as far as he could get. The lump that lives in every tender man's throat jumped up and choked him.

OUR doctor growled for a moment; but his eyes were reading through that other man's distress. "Where do you live?" he asked.

"Carters Corners."

"Carters Corners! Why, that's eight miles from here! How did you come?"

"I walked."

The doctor stared at him more keenly. "Walked! Walked! Why on earth didn't you go to that new doctor who is at Spring Hill? That can't be more than a couple of miles from where you live."

The man with the lantern stood looking dumbly at the floor and had to gulp at the lump three or four times before it would let him speak. "It's our only boy," he said softly, "and one doesn't want to take a chance on strange doctors when it seems like about everything in the world is hangin' on his little life. Everybody says you know most everything, and I came down for you." The big hard fist went up across the man's face and shook as if the strain of waiting for a decision was nearly too much to bear.

The doctor pulled up his coat collar, grumbled because the tired Gray Dick would have to carry two men eight miles, and growled, "Come on!"

"But that man Butcher, he called first," the landlord said, still thinking of the hundred that one would pay compared to what the other could pay.

"If he calls again, tell him I'm out for all night."

"But—"

"There's no 'but' about it! He'll have to wait." He slammed the door behind him, and as he led the way muttered, "A rich man can hire or fire a doctor, or get a new one when he wants to; but a poor man can't. The one he can get looks nearly as big to him as God Almighty."

THEY piled on the horse, the man protesting that he could walk back; but Harvey said he needed him to show the way, and off into the darkness they rode, the big gray plunging wearily but insistently along as if he knew the case was urgent. Harvey's head drooped now and then. His day had been a long one. Once the horse stumbled as if he too was about at the end of endurance. Then they hurried faster, and both horse and man accused themselves of loitering. They came to a little house where the light was shining. The man with the lantern leaped for the door and then, as though fear had grasped his wrist, hesitated.

The doctor shoved him ahead. "Go on," he said, "go on! We can't find anything out by loafing at the door."

They went through to an inner room where the light was. A little girl was asleep at the foot of the bed, and one other, older, stood up as they entered. The mother, a frail woman, was on her knees by the bedside praying and sobbing as though her heart was being torn out by the roots. On the bed lay a boy not more than three years old, his plump baby face red with fever, his eyes opened but unseeing, and his blond curls touseled about his head.

"How long has he been this way?" the doctor asked, leaning far over and looking into the blank pupils.

The mother told him. "Doctor," she implored, "save him! I've lost two! I can't spare no more!"

He felt the pulse and the fingertips in that cool professional way as if estimating the chances, and then turned with a gruff, "Get out of here, all of you!"

He shoved even the sobbing mother from the room, followed, and pulled the door shut after him.

"Now, see here," he admonished, "this is no prayer meeting! We've got to get mighty busy, if we want to keep that boy alive! Got any ice?"

Of course they had not; for even the winter was unkind.

"Any spring near here, a cold one?"

They did have a spring; but it was some way back on the hillside.

"Then get pails, kettles, anything!" Harvey ordered. "Get them quick! Here you, and you, and you, all of you, keep going to that spring and back to this door with cold water! Cold! Do you hear? I want cold water at that door every minute till I say stop! Hurry now! Go on!"

HE went back into the room and closed the door behind him. He wasn't sleepy now, and his eyes had in them the light that comes when determination is so strong that bodily weariness is forgotten. He threw off his coat, vest, and shirt and stood there in his undershirt. He dragged the sheets from the bed and laid the hot little body on the patched quilt.

"Old fellow," he said, "you haven't many chances! You're pretty near the white gate; but I'm going to pull you through unless the Lord wants you more than your mother does!"

A rap at the door, and he reached out for the pail of cold water. He wrung a sheet into its chill depths and deftly wrapped the little boy in its folds. He seized the other sheet, dipped, and wrung, and changed them as fast as he could, muttering to himself as he did so, "This is going to be a long hard session; but if I can only freeze the fever out—Cerebrospinal meningitis, and it's bad!"

Another tap at the door, and a pail was handed in by a frightened little girl, whose way from the spring, in her turn, had been filled with terrors as though Death, which had been lurking round the house, had paused to accompany her. The doctor went steadily on with his work and his grumbling.

"Temperature one hundred and four! It's got to come down! It's got to!"

The perspiration was pouring from him with his exertions and his eyes were half dizzy with fatigue and reiterated movement. Another tap, and a kettle of fresh water was at his hand.

"Temperature one hundred and three! Whoop! Got you going! I've got you!" he roared. And the tousled little head did not move at that strange shout; for in the tiny ears the blood was still drumming in agonizing diapason.

On it went, that splendid fight, minute by minute, and hour by hour. The four water carriers were uncomplainingly following their path in turn, although the mother had wept at her work until the tears would no longer come. The gray swept across the high ridge of the solemn old Schooleys, and the pine trees were silhouetted against the new day.

"No more water for awhile," the doctor whispered through the door to the father. "No more water and—no noise! Keep out!"

Again there was the barrier of the door, blank, impenetrable, and unfeeling. The father and mother, dumb with misery, sat on chairs close by each other as if proximity might mitigate their suffering, and his rough hand crept over and held hers in the eloquence of a terrible silence. The two girls sat side by side on the home-made couch until fatigue overcame them, took toll, and gave them the peace of sleep. The lamp on the table spluttered out, and the daylight, growing stronger, paled its dying flickers. Time itself seemed to have stopped to await the decision. From that inner room the watchers could hear nothing, no enlightening sound.

THE door opened quietly at last, and the doctor came out. He closed it very softly behind him, and when he turned they were on their feet, still holding hands and leaning forward. Something in his attitude chilled them, some suggestion of defeat, some grave look in the tired eyes, some helpless droop of the weary shoulders.

He put his finger to his lips. They could not interpret his meaning. All they knew was that he wanted silence, and whether it was the deferential muteness for the dead or the necessary stillness for the sleeping ill they dared not guess. They met halfway in the room, and for the first time he appeared to comprehend their dread. His shoulders went back to their habitual posture of strength and his eyes glowed with the light of a great victory.

"Your boy will live. He is all right now. I've got his temperature down to nearly normal, and the crisis has passed."

The mother's knees seemed suddenly to give way. She slid to the floor and her arms entwined themselves around his legs while her shoulders twitched convulsively. The father appeared dumb, stupefied, and hopelessly wondering. He stumbled to the ancient clock in the corner, tugged at the glass case, opened it, and took out a knotted handkerchief which he opened and laid on the edge of a rickety table, while the doctor lifted the woman to her feet with a gesture that was half annoyance, half sympathy.

The man with the handkerchief fumbled at it. "I work in the powder mills—laborer, dollar a day," he said in a voice that was still stupefied by relief, "and that's all I've got. There's three dollars and eighty-four cents there."

The doctor came to the table, leaned over, and with his forefinger prodded the tiny heap of coin, which was made up of pennies, nickels, and dimes.

The man watching him misinterpreted his hesitancy for disdain. "It's all I have," he reiterated. "Some day I'll have more and—" his voice died away in a murmur.

The doctor was not thinking of the sum, but of the effort for its accumulation. All they had! All they had saved! How many months had been required to garner those paltry pennies from the dollar a day that had to support five human beings? What sacrifice did it mean? They were paying him their entire fortune, casting it thankfully at his feet and regretting they had no more to give! Not asking favors, mind you, or pleading their poverty, but tendering all they had!

HARVEY picked it up slowly, reverently. It represented so much! The biggest fee he had ever been offered! He tiptoed across the room to the clock from which the hoard had been taken, replaced it, and faced them.

"The child's all right," he whispered. "He'll live." He reached for his hat and put it on his head. He got his overcoat from a hook and donned it unassisted, while the man and woman, motionless and hurt, watched him. He analyzed their looks.

"Listen!" he said with unusual softness and with all the hardness of his manner gone. "This is Christmas morning. I've not had the time to remember it before; but a good many hundred years ago there was another Baby—"

Understanding, they moved toward him as if to try to express their gratitude.

He stiffened instantly to his old brusk form and burst out, "Haven't I a right to make a Christmas present if I want to? I'm giving you your own baby! It's all I could give!"

He fled from the room as if he feared they might read some sympathy, some feminine softness, in his clouded eyes. He was gone. He crawled stiffly into the saddle and turned Gray Dick's head into the mountain trail. The sun suddenly peered at him from across the high hills, and was without its wintry coldness, seeming instead to illumine and warm the world, his world, where he, the country physician, had become in truth a poor man's god. And who knows, could he have but heard, that the air above him was not filled with a finer carol than any proud cathedral might boast on that new broken Christmas Day!

